# Controlling Rotationally Resolved Two-Dimensional Infrared Spectra with Polarization

**DOI:** 10.1021/acs.jpclett.2c03331

**Published:** 2022-12-09

**Authors:** Grzegorz Kowzan, Thomas K. Allison

**Affiliations:** † Department of Chemistry, Stony Brook University, Stony Brook, New York11790-3400, United States; ‡ Institute of Physics, Faculty of Physics, Astronomy and Informatics, Nicolaus Copernicus University in Toruń, ul. Grudzia̧dzka 5, 87-100Toruń, Poland; ¶ Department of Physics and Astronomy, Stony Brook University, Stony Brook, New York11790-3400, United States

## Abstract

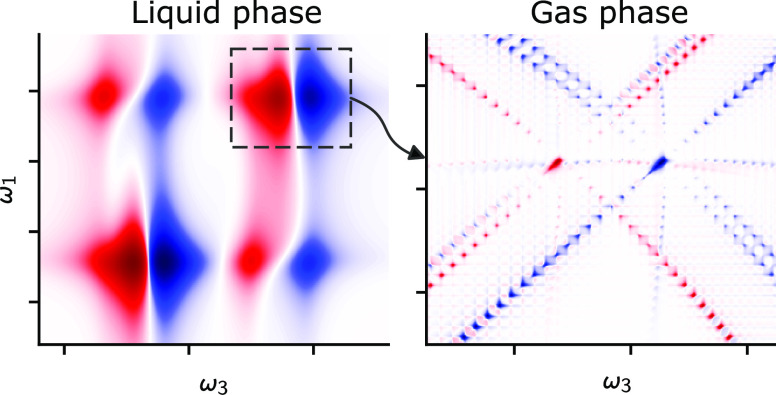

Recent advancements in infrared frequency combs will enable facile recording of coherent two-dimensional infrared spectra of gas-phase molecules with rotational resolution (RR2DIR). Using time-dependent density-matrix perturbation theory and angular momentum algebra techniques, we derive new polarization conditions unique to freely rotating molecules and absent in the condensed phase. These polarization conditions can be used to suppress parts of 2DIR rovibrational response, clarifying complicated RR2DIR spectra. With the polarization control methods described here, RR2DIR spectroscopy can be a powerful tool for studying complex gas mixtures of polyatomic molecules.

Two-dimensional infrared (2DIR) spectroscopy is a nonlinear all-optical technique using ultrashort broadband pulses to study structure and dynamics of molecular systems.^1^ 2DIR methods are commonly used to study liquid- and solid-state samples with large optical densities and broad spectral features, usually using spectrometers with resolution *Δν̅* > 1 cm^–1^. On the other hand, gas-phase systems with narrow spectral features and low optical densities are studied with cavity-enhanced linear spectroscopy^2^ or action-based methods.^3−6^ Despite very low detection limits and high frequency accuracy, high-resolution (*Δν̅* < 0.1 cm^–1^) molecular spectroscopy of gas mixtures^7,8^ remains a difficult problem due to spectral congestion. This issue can be alleviated by adding a second dimension to the spectra.^9^ Sensitive and high-resolution 2DIR spectroscopy in the molecular fingerprint region (λ = 3–20 μm) can enable analysis of complex mixtures of polyatomic gases with unprecedented sensitivity and specificity. These new spectroscopic capabilities would benefit applications in human breath analysis,^8^ flame diagnostics,^10^ and detection of explosives, as well as fundamental chemical physics studies on problems such as intramolecular vibrational redistribution^11^ and collisional dynamics in gases.^10,12,13^ With a large information density of rotationally resolved 2DIR (RR2DIR) spectra, there are likely many unforeseen applications as well.

Recently, several research groups have begun to explore 2DIR spectroscopy of gas-phase molecules. In 2018, Mandal et al.^13,14^ studied the gas-to-liquid transition in supercritical fluids and observed patterns indicative of free rotation but did not observe individual rovibrational resonances. Very recently, Gronborg et al.^15^ presented 2DIR gas-phase spectra of optically thick pure CO_2_ samples at atmospheric pressure. Here, individual rovibrational resonances were resolved but the measured line shapes were dominated by the instrumental line shape rather than the molecular line shape. Recent developments in frequency comb-based methods promise to advance the capabilities for acquisition of RR2DIR spectra much beyond these studies using conventional 2DIR setups based on kHz repetition rate lasers. For example, Allison and co-workers have used frequency comb techniques to enhance ultrafast transient absorption spectroscopy (a third-order response) in dilute gases with detection limits approximately 4 orders of magnitude lower than conventional methods.^16,17^ Allison also framed the more general coherent 2D spectroscopy in terms of wave mixing of multiple frequency combs and described methods for cavity-enhancing 2D spectroscopy signals.^18^ Lomsadze and Cundiff have demonstrated rapid, high-resolution coherent multidimensional spectroscopy in optically thick Rubidium vapors with multiple frequency combs.^19^ In parallel with these technique developments, there has been rapid progress in raising bandwidth and power of mid-IR and long-wave IR frequency comb light sources.^20−25^

Extracting molecular structure and dynamics from condensed phase 2DIR spectra is greatly facilitated by the selection of specific third-order pathways that contribute to the total response. This can be achieved by using different phasematching conditions, phase cycling schemes, and polarization sequences. While most of these tools are directly applicable to RR2DIR spectroscopy, in this Letter we show that the polarization dependence of RR2DIR offers unique opportunities for isolating specific pathways and molecular signatures. We present several new polarization conditions for suppressing certain branches of RR2DIR spectra of freely rotating molecules. The theoretical background enabling derivation of these conditions can be found in ref (26). These polarization conditions are distinct from those commonly used in condensed-phase 2DIR spectra^27,28^ and those previously considered in gas-phase four-wave mixing.^29^ We illustrate the power of these techniques with an example simulation regarding the separation of isotopologues of methyl chloride. The calculations were performed with the accompanying software package rotsim2d.^30^

We consider the case of three ultrashort laser pulses interacting with a gas sample. The third-order polarization is related to the incident field by^1,31^
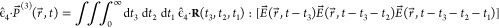
1where *t*_*i*_ are delays between interactions, **R**(t_3_, *t*_2_, *t*_1_) is the third order nonlinear response function, which is a fourth rank tensor, and “:” denotes tensor contraction—here, 3-fold contraction with electric field terms. The unit vector ϵ̂_4_ specifies the polarization detection axis. We consider time-ordered excitation by three ultrashort optical pulses. The direction of the emitted third-order field is specified by *k⃗*_*s*_ = κ_1_*k⃗*_1_ + κ_2_*k⃗*_2_ + κ_3_*k⃗*_3_, and the frequency by ω_*s*_ = κ_1_ω_1_ + κ_2_ω_2_ + κ_3_ω_3_, where *k⃗*_*i*_, ω_*i*_ are wavevectors and frequencies of incident pulses and κ_*i*_ = ± 1. Following common conventions,^32^ we label the directions associated with κ⃗ = (κ_1_, κ_2_, κ_3_) = (−1, 1, 1) as *S*_*I*_ (rephasing) and with (1, −1, 1) as *S*_*II*_ (nonrephasing).

In the impulsive limit (Dirac delta pulses) the third-order signal is given by
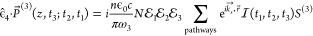
2where the sum is over all experimentally relevant third-order excitation pathways. *N* is the concentration of the active molecule,  are the integrated pulse envelopes, and *S*^(3)^ is the third-order pathway amplitude.^26^ encapsulates molecular dynamics between excitations. Under a simplified model of gas-phase 2D line shapes, individual resonances are represented by complex 2D Lorentzian profiles^1,26^ that mix absorptive and dispersive parts of the response.

Complicated RR2DIR spectra can be clarified by varying the polarization angles of the three incident pulses θ_1_, θ_2_, θ_3_, and of the detected polarization θ_4_. The polarization-dependence of each pathway in eq 2 can be derived using spherical tensor operator techniques,^33,34^ as demonstrated by Williams et al.^35^ and Murdock et al.^29^ The amplitude *S*^(3)^ of each pathway is proportional to an expression depending only on the beam polarizations, **ϵ** = (θ_1_, θ_2_, θ_3_, θ_4_), and on the rotational angular momentum of involved states, *J* = (*J*_*i*_, *J*_*j*_, *J*_*k*_, *J*_*l*_), given by^26,29^
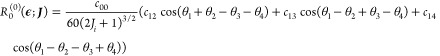
3where the *c*_*αβ*_ coefficients in general depend on *J*, but in the limit of high *J*_*i*_—valid for *J*_*i*_ ≳ 10—they only depend on differences between *J*_α_ values.^26,29^ In ref (26), we separated all rovibrational third-order pathways into 7 classes with regards to their dependence on the polarization angles in the high-*J*_*i*_ limit. Here we present three new polarization conditions derived using this theory that suppress whole branches of third-order pathways and greatly simplify RR2DIR spectra.

We illustrate the structure of RR2DIR spectra and the effect of our new polarization conditions in Figure 1. The figure shows simulated spectra of the methyl chloride ν_3_ mode, produced by summing all rovibrational pathways starting in the ground vibrational state with *J* values up to 30. This and subsequent simulations were performed with our rotsim2d code^30^ using molecular data from the HITRAN database.^36,37^ The figure shows *t*_2_-dependent 2D resonance amplitudes:
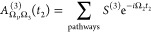
4For clarity, this quantity does not include the full 2D lineshapes. Panels a and f show the total molecular response with all polarizations aligned. The Ω_2_ frequency is nonzero for rotationally coherent (RC) pathways and imparts *J*_*i*_-dependent phase on associated resonances. This is seen as modulation along antidiagonal branches for rephasing (a) and along diagonal branches for nonrephasing (f) spectra. The “x”-shaped pattern is produced by P- or R-type coherences oscillating during *t*_1_ and *t*_3_ times. For example, the lower left quadrant contains negative P–P signal and positive P–2P signal, whereas the upper left quadrant contains P–R and P–2R signals. The fainter “+”-shaped pattern is produced by Q-type coherence evolution during *t*_1_ and P- or R-type coherence during *t*_3_ or *vice versa*. For example, the almost horizontal branches in the left half are due to Q–P and Q–2P resonances. See the ref (26) for a more detailed description of the branch structure of RR2DIR spectra.

**Figure 1:**
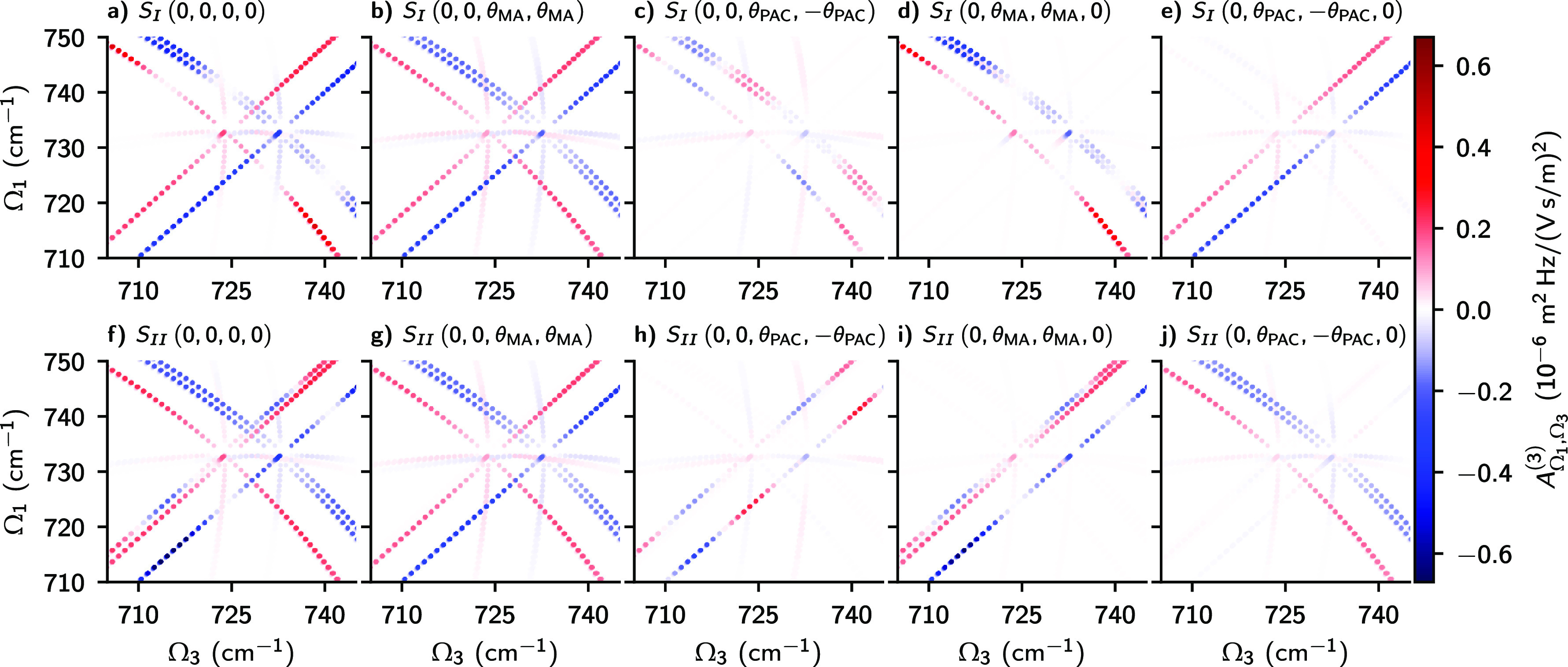
Effect of different polarization conditions on 2D resonance amplitudes, *A*_Ω_1_,Ω_3__^(3)^(*t*_2_ = 1 ps). Modulation along the branches is caused by rotationally coherent pathways. The leftmost column, with panels a and f, presents the pathway amplitudes for beam and detection polarizations all aligned. The remaining panels show the effects of four special polarization conditions on *S*_*I*_ and *S*_*II*_ pathways.

A well-known polarization condition in nonlinear spectroscopy is the so-called “magic angle” (MA) condition where pump and probe pulses have an angle of θ_MA_ = tan^–1^ √2 between them, i.e., **ϵ** = (0, 0, θ_MA_, θ_MA_). The effect of the magic angle condition is often treated classically.^1^ In the context of 2DIR spectra with resolution of rotational eigenstates, the standard MA condition is more easily understood as a condition that eliminates RC Feynman pathways, as shown in panels b and g of Figure 1. The standard MA condition offers some control of RR2DIR spectra and can help with spectral congestion, but all main branches remain intact.

For more control of the spectrum, we introduce several new polarization conditions whose effects are illustrated in panels c–e and panels h–j of Figure 1. For these, we introduce the population-alignment canceling (PAC) angle
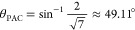
5The arrangement (0, 0, θ_PAC_, −θ_PAC_), illustrated in Figure 1, parts c and h suppresses the non-RC antidiagonal and diagonal branches involving P- and R-type coherences, but it leaves unaffected branches involving Q-type coherences. As a result, this condition brings out the RC pathways in the spectrum and gives a strongly *t*_2_-dependent signal. Another condition we introduce is the middle MA condition, (0, θ_MA_, θ_MA_, 0), which has some similarities to (0, 0, θ_PAC_, −θ_PAC_), but differs in details. For *S*_*I*_ (Figure 1d), the middle MA condition suppresses off-diagonal branches with Q-type coherences and the diagonal branches, leaving only the antidiagonal ones and the central Q–Q and Q–2Q branches. For *S*_*II*_ (Figure 1i), the effect of this condition is to remove all nondiagonal branches. A third new arrangement, the middle PAC condition (Figure 1e,j) achieves the opposite of the middle MA condition. It suppresses the antidiagonal resonances for *S*_*I*_ and the diagonal branches for *S*_*II*_. As we show in the examples below, using these new polarization conditions can offer dramatic benefits when dealing with RR2DIR spectra of multiple species.

We expect our polarization conditions to bring most benefits to highly congested spectra, but to clearly demonstrate their effects we present a simulation of a simple two-component mixture. In Figure 2 we plot 2D spectra (top panels) and ω_1_ = ω_3_ line outs (bottom panels) of a mixture of 50% CH_3_^35^Cl and 50% CH_3_^37^Cl under different polarization conditions. The plotted quantity is the third-order amplitude,
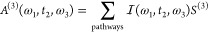
6where  is a partial Fourier transform of  see eq 2. The improvement in selectivity simply due to acquiring 2D IR instead of linear spectra is shown in panel a, which shows standard absorptive 2D spectrum under the MA condition. The diagonal line out (green curve) is highly congested, since it mostly reproduces the features of linear absorption spectrum (black curve). On the other hand, the antidiagonal branches are reasonably well separated and allow us to easily identify components of the signal associated with different isotopologues. Nevertheless, resonances coming from the same or different isotopologues overlap over significant regions of the spectrum. Clearly, this effect will become more pronounced in more complicated mixtures, which will make it more difficult to identify all the species and to determine their concentrations by fitting the spectrum. This problem is solved and the spectral overlap is minimized when using our middle MA condition, which removes the “+”-shaped pattern and non-Q-branch diagonal signals, as shown in panel b. Moreover, as long as Q branches do not overlap, different species can be identified by only acquiring the ω_1_ = ω_3_ cut of the spectrum, as seen in the bottom panel.

**Figure 2:**
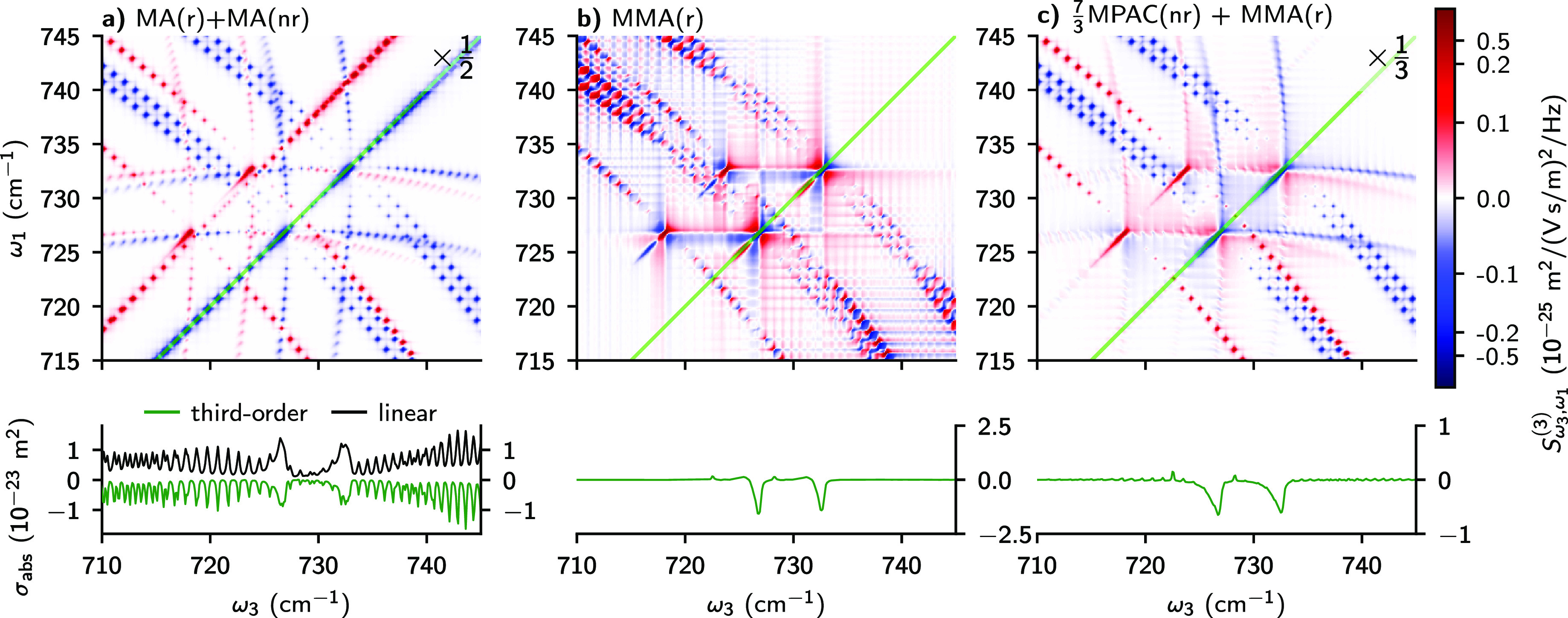
Spectra of 50% of CH_3_^35^Cl and 50% of CH_3_^37^Cl at *T* = 296 K and pressure of 1 atm. Top panels are full 2D spectra and the green curves in bottom panels are diagonal line outs along the green lines. Logarithmic scale is used for |*S*_ω_3_,ω_1__^(3)^| > 0.16 and linear scale for lower absolute values. (a) 2D absorptive spectrum under the magic angle (MA) condition at *t*_2_ = 1 ps. Amplitude is scaled by ^1^/_2_ to match common intensity scale. Black curve in the bottom panel is the linear absorption spectrum. (b) Rephasing spectrum under the middle magic angle (MMA) condition at *t*_2_ = 1 ps. (c) Sum of ^7^/_3_ of nonrephasing spectrum under the middle PAC (MPAC) condition and of rephasing spectrum under the middle magic angle (MMA) condition. Both spectra evaluated at *t*_2_ = 0. Amplitude is scaled by ^1^/_3_ to match common intensity scale.

A potential disadvantage of our condition in Figure 2b compared to the standard 2D IR scheme in Figure 2a is that the former probes broad complex 2D Lorentzian shapes. Better signal separation can be obtained by recording purely absorptive 2D spectra as in Figure 2a on top of suppressing the diagonal and “+”-shaped branches. We can combine the desirable features of both schemes by adding spectra obtained under two of our polarization conditions. The appropriate sum of scaled nonrephasing spectrum under the middle PAC condition and rephasing spectrum under the middle MA condition is shown in Figure 2c. In this case, there is a residual “+”-shaped pattern of nonrephasing resonances, but the remaining antidiagonal features are purely absorptive and the diagonal is still empty of non-Q-branch resonances. In the special case of diatomic molecules or stretching modes of polyatomic linear molecules, purely absorptive spectra of only diagonal or antidiagonal resonances can be obtained by a different combination of polarization conditions. Absorptive antidiagonal spectra are obtained by a sum of nonrephasing spectrum under the middle PAC condition and of rephasing spectrum under the alternating PAC condition, introduced in ref (26). On the other hand, absorptive diagonal spectra are obtained by a sum of nonrephasing spectrum under the alternating PAC condition and of rephasing spectrum for the middle PAC condition.

Lastly, we estimate experimental detection limits for a standard pump–probe geometry measurement with a Fourier-transform spectrometer. We assume Fourier transform-limited Gaussian pulses with duration of 300 fs and bandwidth of 50 cm^–1^, producing frequency combs with a repetition rate of 100 MHz. We set the average power of pump beams at 2 W and assume beam radii of 250 μm. At *t*_1_ = *t*_2_ = 0, 1 atm of pure methyl chloride, *T* = 296 K, and a path length of 1 cm, the peak pump-induced change in the probe absorbance is 1.7 × 10^–2^. Assuming an average probe power of 1 mW and resolution of 1 GHz, the shot-noise-limited, single-element noise-equivalent concentration is  As demonstrated for transient absorption spectroscopy,^16,17^ the detection limits can be improved by >10^4^ to reach the  level by using the cavity enhancement schemes proposed in the literature.^18^

In summary, RR2DIR spectroscopy combined with the presented polarization conditions has the potential to become a valuable investigative tool in fundamental science and applications. The presented polarization conditions are also directly applicable to asymmetric top molecules. Since whole branches are affected, high resolution is not required to benefit from them. In fact, their effect on spectra would be particularly illuminating in high-pressure studies of line mixing^10^ and the gas-to-liquid transition.^13^ It is feasible to obtain detection limits suitable for trace-gas detection, including investigation of vibrational dynamics in cold molecular beams,^17^ applications in breath analysis,^8^ combustion,^10^ and plasma science.^38^
